# Determinants of Use of Household-level Water Chlorination Products in Rural Kenya, 2003–2005

**DOI:** 10.3390/ijerph7103842

**Published:** 2010-10-25

**Authors:** Amy E. DuBois, John A. Crump, Bruce H. Keswick, Laurence Slutsker, Robert E. Quick, John M. Vulule, Stephen P. Luby

**Affiliations:** 1 Enteric Diseases Epidemiology Branch, Division of Foodborne, Bacterial and Mycotic Diseases, National Center for Zoonotic, Vectorborne, and Enteric Diseases, Centers for Disease Control and Prevention, 1600 Clifton Rd., Atlanta, GA 30333, USA; E-Mails: jcrump@cdc.gov (J.A.C.); RQuick@cdc.gov (R.E.Q.); sluby@icddrb.org (S.P.L.); 2 Epidemic Intelligence Service, Career Development Division, Office of Workforce and Career Development, Centers for Disease Control and Prevention, 1600 Clifton Rd., Atlanta, GA 30333, USA; 3 Procter & Gamble Health Sciences Institute, Procter & Gamble Plaza, Cincinnati, OH 45201, USA; E-Mail: Keswick.bh@pg.com (B.H.K.); 4 CDC/Kenya Medical Research Institute, Mumias Rd., Kisian, Nyanza Province, Kenya; E-Mails: lms5@cdc.gov (L.S.); JVulule@kisian.mimcom.net (J.M.V.)

**Keywords:** water, point-of-use, chlorination

## Abstract

Household-level water treatment products provide safe drinking water to at-risk populations, but relatively few people use them regularly; little is known about factors that influence uptake of this proven health intervention. We assessed uptake of these water treatments in Nyanza Province, Kenya, November 2003–February 2005. We interviewed users and non-user controls of a new household water treatment product regarding drinking water and socioeconomic factors. We calculated regional use-prevalence of these products based on 10 randomly selected villages in the Asembo region of Nyanza Province, Kenya. Thirty-eight percent of respondents reported ever using household-level treatment products. Initial use of a household-level product was associated with having turbid water as a source (adjusted odds ratio [AOR] = 16.6, *p* = 0.007), but consistent usage was more common for a less costly and more accessible product that did not address turbidity. A combination of social marketing, retail marketing, and donor subsidies may be necessary to extend the health benefits of household-level water treatment to populations most at risk.

## 1. Introduction

According to the United Nations Children’s Fund [1], only 46% of the population of Kenya has access to improved water sources. Since not all water from improved sources meets World Health Organization (WHO) guidelines for potable water and since access to improved water may be intermittent, an even higher percentage do not have consistent access to safe water [2,3]. In rural Kenya, where there has been slow progress toward improved water systems, [4,5] people have another option for obtaining safe water. Household-level water treatment products offer an immediate, affordable alternative to resource-intensive networked systems for providing safe drinking water for Kenyans and millions of others throughout the developing world. While the health benefits of household-level products are well-documented [6–9], motivating consistent use remains a significant challenge [10].

Nyanza Province in western Kenya is among the poorest regions in Kenya with 2.4 million people or 64% of the population living below the poverty line [11]. The vast majority of homes are not equipped with electricity, few communities have even public taps, and all lack sewerage systems. Water is often collected daily from ponds and rivers where livestock also drink and stored in the family compounds in 10–20 liter clay or plastic containers. Water from these sources is often highly turbid due to organic sediments and contaminated with enteric pathogens.

Products for household-level treatment of drinking water are available in the area. Locally-produced sodium hypochlorite solution (Jet Chemicals, Ltd, Kenya) has been socially marketed since May, 2003 and a flocculent-disinfectant product was introduced later that year. Developed and manufactured by the Procter & Gamble Company (Ohio, United States of America) when the flocculent-disinfectant is mixed with highly turbid water, debris quickly settles and the water becomes visibly clear and disinfected. Highly turbid water has high chlorine demand, and previous research has demonstrated the ability of the flocculent-disinfectant to render such water potable [12]. The locally produced sodium hypochlorite solution is a highly effective disinfectant under most conditions, but functional chlorine concentration, and the odor and taste of treated water, can be compromised by the organic materials in highly turbid water. Unlike the flocculent-disinfectant product, sodium hypochlorite solution does not improve the clarity of highly turbid water. A health outcomes study in Kenya demonstrated a general reduction in diarrhea for households using either household-level product *versus* traditional untreated water handling methods and a statistically significant 25% reduction in diarrhea among children < 2 years in compounds using flocculent-disinfectant compared to traditional untreated water handling methods [13]. Despite the benefits [9], studies have demonstrated that even the experience of decreased diarrheal disease burden is not adequate to motivate consistent behavior change [10]; clearly there are other factors at play.

**Figure 1.**
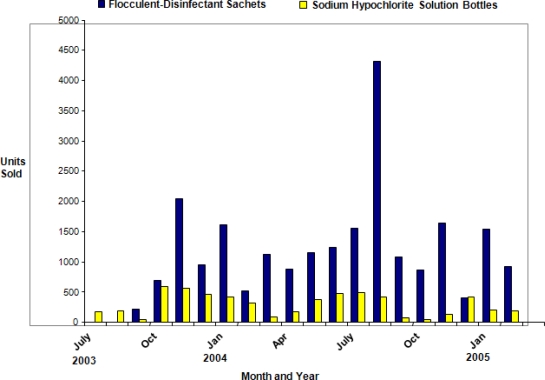
Monthly Sales of Flocculent-disinfectant Sachets and Sodium Hypochlorite, Asembo and Gem sub-districts, Nyanza, Kenya. *August 2004 totals include bulk sale of flocculent-disinfectant to NGO; **One sachet of flocculent-disinfectant treats 10 liters and one bottle of sodium hypochlorite treats 2,500 liters of water. Flocculent-disinfectant costs 10 Ksh per 20 L water treated; sodium hypochlorite costs 0.4 Ksh per 20 L water treated.

We hypothesized that the immediate reinforcement of visibly clear water would be a strong motivator for use of flocculent-disinfectant product. Understanding motivators and identifying successful distribution models for water treatments products could enhance uptake of household-level water treatment and increase the numbers of persons receiving the benefits of safe water worldwide. In 2003, a local non-governmental organization, the Society for Women and AIDS in Kenya (now known as the Safe Water and AIDS Project, or SWAP), began selling the flocculent-disinfectant in Asembo and Gem (subdistricts of Bondo and Siaya Districts, respectively). Social marketing of sodium hypochlorite solution began in the area in 2000 and SWAP began campaigns for the flocculent disinfectant beginning in 2003 after the health outcomes study introduced the product to the community. Campaigns included training and mobilization with community groups, presentations, distribution of educational materials, and micro-finance projects. In one pilot micro-enterprise project, local individuals and community groups had the opportunity to purchase quantities of the product and sell it in their villages at a small margin over wholesale cost. SWAP intensified activities starting in May 2003 and integrated the flocculent-disinfectant into its community education campaigns that already promoted the sodium hypochlorite solution with safe water storage and other health-related behaviors. From November 2003–February 2005, we conducted three studies to assess usage patterns of these two water treatment products and document use-prevalence in Asembo, Kenya. Figure 1 provides details on sales volume of the two products during the study period.

The three studies included: (1) a baseline utilization study (November 2003); (2) a follow-up utilization study (January 2005); (3) a use-prevalence survey for water treatment products in the study area (February 2005). Campaigns related to household water treatment products were ongoing throughout this time period.

## 2. Experimental Section

### 2.1. Baseline Utilization Study (2003)

#### 2.1.1. Study Design

The study assessed characteristics of persons who used the newly-available flocculent-disinfectant water treatment product. We defined a user as a person living in Asembo who purchased and used any quantity of the flocculent-disinfectant product to treat water for the family compound. Users were identified by review of records from SWAK and flocculent-disinfectant vendors. Non-user controls were randomly selected from family compounds in Asembo within 1 kilometer of one of the eight flocculent-disinfectant vendors using spatial mapping and census data from the CDC/KEMRI Demographic Surveillance System (DSS). After consent was obtained from the head of the family compound, interviews were conducted at each compound with the mother of the youngest child in the compound. All respondents answered questions about beliefs concerning water and diarrheal diseases, drinking water sources, water treatment and storage practices, familiarity with water treatment products, and indicators of socioeconomic status such as educational level, cash spending on hygiene products, housing characteristics and household goods. Researchers documented the presence or absence of soap, toothpaste, and water treatment products in each compound. Stored household water was tested for residual free chlorine using the N, N-diethyl-phenylenediamine colorimetric method (Colorwheel Chlorine Test Kit, Hach® Company, Loveland, CO).

#### 2.1.2. Analysis

Data were entered into an Access® database with the Cardiff TELEform® image scanning system (Autonomy Cardiff Corporation, Vista, CA). Analysis was performed using STATA10 (Stata Corporation, College Station, TX). A socioeconomic status index was constructed using principal components analysis in the manner described by Vyas and Kumaranayake [14]. Bivariate analysis included two-sided Student’s t-test of means for continuous variables and Fisher’s exact test for categorical variables. Factors that were statistically significant at *p* < 0.05 were included in a multivariate model. We developed a multivariate logistic regression model to identify independent associations with use of the flocculent-disinfectant. Covariates and interaction terms were tested for significance and goodness-of-fit. Model checking was performed using likelihood ratio testing.

### 2.2. Follow-up Utilization Study (2005)

#### 2.2.1. Study Design

Family compounds of flocculent-disinfectant users who participated in the 2003 utilization study were revisited. Efforts were made to locate the same person who was interviewed in 2003. Participants answered 64 standard questions regarding drinking water sources, water storage and treatment, and socioeconomic indicators for the household. Observers documented the presence of nine water treatment and hygiene items such as soap. Stored household water was tested for the presence of chlorine using a standard pool test kit (Aquality Professional Duo-Test, STA-RITE Industries, Delavan, WI). We defined reported consistent use based on number of sachets purchased relative to water consumption and conducted a separate analysis on the sub-group with confirmed use based on the presence of chlorine in the household drinking water at the time of the interview.

#### 2.2.2. Analysis

Statistical analysis was completed using STATA10 and the methods described for the baseline study.

### 2.3. Use-prevalence Survey for Water Treatment Products (2005)

#### 2.3.1. Study Design

This study documented use-prevalence for household-level water treatment products in the study area. We randomly selected 10 villages from Asembo. Flocculent-disinfectant had been available for sale since November 2003 in each of these villages and the surrounding area. An interviewer and a village health worker, using the most recent DSS census, visited all compounds in these villages. A person in the compound with responsibility for water handling answered four questions regarding household-level water treatments in the previous 7 days and since the short rains of 2003 (November–December 2003).

#### 2.3.2. Analysis

Use-prevalence was calculated for flocculent-disinfectant and sodium hypochlorite during the two time periods.

## 3. Results and Discussion

### 3.1. Baseline Utilization Study

We enrolled 117 persons who met the definition of flocculent-disinfectant user and 193 control-persons who had never used the flocculent-disinfectant (Table 1). Flocculent-disinfectant users were more likely to use a turbid water source (Odds Ratio [OR] = 19.7, 95% Confidence Interval [CI] = 3.1–812) and to attribute diarrhea to their drinking water (OR = 2.5, CI = 1.4–4.6). Users were less likely to express the belief that diarrhea is a serious problem in the community (OR = 0.4, CI = 0.3–0.7). Mean spending on soap and toothpaste was significantly higher for users *versus* non-users (46.5 *versus* 37.2 Ksh, *p* = 0.02). The mean socioeconomic status index was significantly higher for users than non-users (*p* = 0.001).

After adjustment for economic status index, spending on soap and toothpaste, and knowledge of the previous CDC/KEMRI study, two factors remained significantly associated with flocculent-disinfectant use. Use of turbid water sources was strongly associated with flocculent-disinfectant use (Adjusted Odds Ratio [AOR] = 19.7 CI = 2.5–153; *p* = 0.004). Those who used flocculent-disinfectant remained less likely to express the belief that diarrhea is a serious problem in the community (AOR = 0.4, CI = 0.3–0.7; *p* = 0.001).

### 3.2. Follow-up Utilization Study

Of the 117 users in the baseline utilization study, 104 (89%) completed questionnaires for the follow-up study. (Table 2) Of those interviewed, eight (8%) reported using flocculent-disinfectant in the past 7 days. Twenty-six (25%) had not used the flocculent-disinfectant since the time of the baseline study. Overall, 50 (48%) reported treating their water by some method in the past 7 days. Of those who did not use the flocculent-disinfectant consistently, the most commonly cited reasons were lack of availability (66%) and expense (20%). Of the 78 (75%) respondents reporting flocculent-disinfectant use since the study period in December 2003, 65 (83%) purchased the flocculent-disinfectant directly from a SWAP representative and only 11 (14%) reported purchase from a *duka* (small shop). In contrast, of the 74 (71%) respondents who reported use of sodium hypochlorite in that time same period, 37 (47%) reported purchase from a *duka* and 20 (26%) reported purchase from a market.

Although 18 (17%) respondents reported daily use of either the flocculent-disinfectant or the sodium hypochlorite solution on the questionnaire, only 14 reported chlorinating the water stored in their home at the time of the interview; 11 of these 14 (11% of 104 total respondents) had free chlorine present in their stored water.

On bivariate analysis, drinking water from turbid sources at least 4 months per year was reported by 96 (92%) respondents. Socioeconomic status was not associated with reported consistent use (OR = 0.9, CI = 0.7–1.3; *p* = 0.6).

On multivariate analysis, after adjusting for economic status and awareness of the previous CDC/KEMRI study, respondents who reported consistent use were less likely than reported sporadic users to express the belief that their drinking water made their family sick (AOR = 0.34, CI = 0.1–0.9; *p* = 0.03). Socioeconomic status was not significantly associated with reported consistent use after adjustment for these other factors. These associations were essentially unchanged regardless of whether reported consistent use was defined by reported volume of flocculent-disinfectant used or by confirmation of presence of free chlorine in the household water at the time of the interview.

### 3.3. Use-Prevalence Survey

Of the 1,530 compounds listed in the most recent DSS census of Asembo, a total of 1,452 (95%) were included in the survey. Five-hundred-thirty-one (37%) compounds reported ever using the sodium hypochlorite solution compared with 105 (7%) who reported ever using the flocculent-disinfectant. Two-hundred-twenty-four (15%) reported use of the sodium hypochlorite in the past 7 days while 14 (1%) reported using the flocculent-disinfectant in that time period. Overall, 549 (38%) compounds reported ever using some form of household-level water treatment and 231 (16%) reported household-level water treatment in the past 7 days. Village-specific rates for ever using the flocculent-disinfectant varied from 0.7% to 16%. Rates for use of flocculent-disinfectant the past 7 days ranged from 0% (6 villages) to 13%. Reports of ever using the sodium hypochlorite solution ranged from 21% to 59%, while rates of use in the past 7 days ranged from 7% to 27%.

These studies demonstrate a complex array of issues contributing to use of household-level water treatment products in western Kenya. While initial use of the flocculent-disinfectant was strongly associated with having turbid drinking water, this association did not persist in the study of reported consistent use. Although cost is often cited anecdotally as a reason for lack of use of household-level water treatment products, in our study economic status was not associated with reported consistent use among early users. Improvements in health do not seem to definitively influence use either: Luby *et al.* have demonstrated that even the experience of decreased diarrheal disease burden among residents of rural Guatemala was not adequate to motivate consistent use [10].

Dependence on a turbid water source emerged as the strongest motivator for flocculent-disinfectant use in this setting. The association with turbidity persisted after adjusting for socioeconomic status, spending on personal care items, and beliefs about the relationship between water and health. This result supports the hypothesis that the ability of flocculent-disinfectant to visibly clear turbid water is a compelling impetus to initial use. However, the allure of clearer water was not associated with reported consistent use based on the data from the follow-up survey. In this cohort with prior experience with flocculent-disinfectant and a high dependence on turbid water, sporadic use of sodium hypochlorite solution was comparable to use of the flocculent-disinfectant (71% *versus* 78%). The relatively high use of sodium hypochlorite despite the turbid water burden may be a reflection of familiarity with the sodium hypochlorite solution. Lower cost or greater ease of use for sodium hypochlorite may also have been determinants of use despite the advantages of flocculent-disinfectant for those using tubid water. Our data suggest that consumers often tried both locally available products, but reported using sodium hypochlorite more consistently than flocculent-disinfectant, for both the past year and the past week. Seventy-five percent of those who used the flocculent-disinfectant since 2003 also used sodium hypochlorite solution in that time period. In both the community as a whole and among those who used flocculent-disinfectant at baseline, the prevalence of sodium hypochlorite use eventually surpassed flocculent-disinfectant use. Thus, although dependence on turbid water correlated with trying flocculent-disinfectant, other factors appear to influence the decision to treat household water consistently and what product to use for this treatment. Since the time of the study, flocculent-disinfectant has expanded to national distribution networks in Kenya; this expansion may increase use by addressing the issues of availability that we found in our study.

Economic factors clearly influenced usage patterns. The choice of sodium hypochlorite over flocculent-disinfectant may largely be a function of the difference in retail cost as sodium hypochlorite solution cost less than 1 US cent per 20 L of water treated while flocculent-disinfectant cost 12 US cents per 20 L treated. Use did in fact decline remarkably in the follow-up survey with 25% of initial users reporting they never used the flocculent-disinfectant product again; however, the lack of a statistically significant relationship between reported consistent use and socioeconomic status in the follow-up survey suggests that something besides finances also affects usage patterns. The manufacturer is undertaking price-reduction studies in rural Kenya for the flocculent-disinfectant; these may provide a sense of how much affordability ultimately impacts use.

Lack of availability emerged as an important determinant of flocculent-disinfectant use based on data from the cohort of prior users. Based on qualitative data from interviews, problems with flocculent-disinfectant distribution caused gaps in availability that in turn pre-empted use. Availability of flocculent-disinfectant in the local market decreased dramatically after the change in credit policy at SWAP. Rural community groups who served as vendors during the initial phase of sales did not have adequate cash resources to purchase flocculent-disinfectant in bulk. Without income generation from the wholesale purchases, these groups could not sustain the retail market. Those who reported buying sodium hypochlorite reported purchases from multiple sources including *dukas*, markets and chemist shops. These locales are part of the indigenous consumer culture, and availability there made sodium hypochlorite much more accessible than flocculent-disinfectant, which had minimal penetration into these venues. Further penetration into the conventional retail sector may contribute to increased use of the flocculent-disinfectant through more consistent availability.

The documented prevalence of nearly 40% for ever using household-level water treatment products in this rural Kenyan setting demonstrates their potential as a way for even severely economically disadvantaged persons to benefit from safe water. The challenge lies in getting households to adopt this proven intervention. Behavior change communication can help; teaching safe water handling in elementary schools and clinics has demonstrated increased household use of water treatment products in pilot studies [15,16]. These factors may not be sufficient motivation if prices are too high. In our context, micro-credit programs through an NGO made it possible for communities to purchase stock at wholesale prices thus making the products accessible to more people. In another study in western Kenya, Freeman *et al.* found that although awareness of household-level water treatment products was high across wealth quintiles, use dropped precipitously in the lowest quintile [17]. In the poorest segments of the population, where morbidity and mortality from waterborne diseases are highest, consistent use of either household-level water treatment product may require subsidies outside of the retail market for the foreseeable future.

Inspiring sustained use will require consistent availability, affordability in the local context, and a more comprehensive understanding of the factors that motivate those who consistently treat their water. This understanding will require further research and data-driven implementation strategies that address the behavioral and economic issues along with the public health issues. Such strategies could be informed by more in-depth behavioral research to further explain the behaviors and choices documented in our studies and specifically assess the relationships between use and social marketing activities. The World Health Organization’s International Network to Promote Household Water Treatment and Safe Storage, a collaboration of UN agencies, bilateral development agencies, international non-governmental organizations, research institutions, international professional associations, the private sector, and industry associations provides an integrated forum for identifying research needs on household-level water treatment and informing policies and programs [18].

The study was limited by several factors. Low prevalence of usage in the community made it difficult to determine robust statistical associations for factors affecting flocculent-disinfectant use. Small sample size also prevented comparisons between those who used various combinations of water treatments, and analysis of seasonality of use; however, the sample sizes were sufficient to identify major factors associated with use.

Courtesy bias likely resulted in some over-reporting of use, based on the results of the utilization study in which 16% of those reporting chlorination did not test positive for residual free chlorine. In addition, cross-sectional studies do not permit an objective assessment of consistent use.

## 4. Conclusions

Household-level water treatment offers an immediate method for providing safe water to millions of people who will not have access to improved water delivery systems in the foreseeable future. These benefits cannot be realized without a better understanding of factors motivating use of the products. To increase usage of household-level water treatment in western Kenya, treatment products must first be consistently available at prices at risk-populations can afford. Availability in the traditional retail sector and through non-traditional vendors will maximize consumer access. NGOs play an important role in generating a consumer impulse for household-level water treatment products through community education and social marketing, but consistent use may require ongoing cost subsidies if the products are to reach those who need them most. The target population without access to adequate water infrastructure is generally the population with minimal financial resources. Visible clearing of turbid water and concern about waterborne diseases drive usage to some extent, but more complex factors appear to ultimately determine selection and consistent use of household-level water treatment products. If household-level treatment products are to fulfill their potential for improved health through safe water, multi-disciplinary implementation programs will need to address both the key barriers of access and affordability and the more nuanced challenge of positive behavior change.

## Figures and Tables

**Table 1.** Baseline Utilization Study: Selected Characteristics of Flocculent-Disinfectant Users, November–December, 2003.

**Characteristic**	**Flocculent Disinfectant Users N = 117 (%)**	**NonUsers N = 193 (%)**	**Crude Odds Ratio**	CI*
Turbid water source	116 (99)	165 (86)	19.7	3.1–812
Believe water quality a problem	116 (99)	190 (98)	1.8	0.1–97
Attribute diarrhea to drinking water	97 (83)	128 (66)	2.5	1.4–4.6
Believe water makes family sick	92 (79)	114 (59)	2.6	1.5–4.5
Believe diarrhea is a serious problem	48 (41)	122 (63)	0.4	0.3–0.7
Have knowledge of the CDC/KEMRI Turbid Water Study March–Oct 2003	82 (70)	85 (44)	3.0	1.8–5.0

**Table 2.** Characteristics Users of Sodium Hypochlorite and Flocculent-Disinfectant, Kenya 2005.

**Characteristic**	**Sodium Hypochlorite n (%)**	**Flocculent-Disinfectant n (%)**

Proportion of water treated in household regularly		

All	26 (25)	29 (28)
Some	35 (34)	48 (46)
None	43 (41)	26 (25)

Used during past year	74 (71)	78 (75)

Used in past 7 days	39 (38)	8 (8)

Where purchased		

SWAK rep (field)	65 (71)	34 (44)
Friend/Neighbor	18 (20)	15 (19)
Duka/Shop/Chemist	11 (12)	37 (47)
Stopped SWAK vehicle	9 (10)	2 (3)
Market	5 (6)	20 (26)
SWAK Office	4 (4)	1 (1)
